# Time for a paradigm shift in shared decision-making in trauma and emergency surgery? Results from an international survey

**DOI:** 10.1186/s13017-022-00464-6

**Published:** 2023-02-17

**Authors:** Lorenzo Cobianchi, Francesca Dal Mas, Vanni Agnoletti, Luca Ansaloni, Walter Biffl, Giovanni Butturini, Stefano Campostrini, Fausto Catena, Stefano Denicolai, Paola Fugazzola, Jacopo Martellucci, Maurizio Massaro, Pietro Previtali, Federico Ruta, Alessandro Venturi, Sarah Woltz, Haytham M. Kaafarani, Tyler J. Loftus, the Team Dynamics Study Group, Recayi Aapoäÿlu, Kenneth Lyle Abbott, Abubaker Abdelmalik, Nebyou Seyoum Abebe, Fikri Abu-Zidan, Yousif Abdallah Yousif Adam, Harissou Adamou, Dmitry Mikhailovich Adamovich, Ferdinando Agresta, Antonino Agrusa, Emrah Akin, Mario Alessiani, Henrique Alexandrino, Chiara Bidoli, Syed Muhammad Ali, Vasilescu Alin Mihai, Pedro Miguel Almeida, Mohammed Mohammed Al-Shehari, Michele Altomare, Francesco Amico, Michele Ammendola, Jacopo Andreuccetti, Elissavet Anestiadou, Alfredo Annicchiarico, Amedeo Antonelli, Daniel Aparicio-Sanchez, Antonella Ardito, Giulio Argenio, Catherine Claude Arvieux, Catherine Arvieux, Ingolf Harald Askevold, Boyko Tchavdarov Atanasov, Goran Augustin, Selmy Sabry Awad, Giulia Bacchiocchi, Carlo Bagnoli, Hany Bahouth, Efstratia Baili, Lovenish Bains, Gian Luca Baiocchi, Miklosh Bala, Carmen Balaguà©, Dimitrios Balalis, Edoardo Baldini, oussama Baraket, Suman Baral, Mirko Barone, Alberto Gonzãlez Barranquero, Jorge Arturo Barreras, Gary Alan Bass, Zulfu Bayhan, Giovanni Bellanova, Offir Ben-Ishay, Fabrizio Bert, Valentina Bianchi, Helena Biancuzzi, Raluca Bievel Radulescu, Mark Brian Bignell, Alan Biloslavo, Roberto Bini, Daniele Bissacco, Paoll Boati, Guillaume Boddaert, Branko Bogdanic, Cristina Bombardini, Luigi Bonavina, Luca Bonomo, Andrea Bottari, Konstantinos Bouliaris, Gioia Brachini, Antonio Brillantino, Giuseppe Brisinda, Maloni Mamada Bulanauca, Luis Antonio Buonomo, Jakob Burcharth, Salvatore Buscemi, Francesca Calabretto, Giacomo Calini, Valentin Calu, Fabio Cesare Campanile, Riccardo Campo Dall’Orto, Andrea Campos-Serra, Joao Miguel Carvas, Marco Cascella, Gianmaria Casoni Pattacini, Valerio Celentano, Danilo Corrado Centonze, Marco Ceresoli, Dimitrios Chatzipetris, Antonella Chessa, Maria Michela Chiarello, Mircea Chirica, Serge Chooklin, Christos Chouliaras, Sharfuddin Chowdhury, Pasquale Cianci, Nicola Cillara, Stefania Cimbanassi, Stefano Piero Bernardo Cioffi, Enrique Colãis-Ruiz, Elif Colak, Luigi Conti, Alessandro Coppola, Tiago Correia de Sa, Silvia Dantas Costa, Valerio Cozza, Giuseppe Curro’, Kirsten Felicia Ann-Sophie Aimee Dabekaussen, Fabrizio D’acapito, Dimitrios Damaskos, Giancarlo D’Ambrosio, Koray Das, Richard Justin Davies, Andrew Charles de Beaux, Sara Patricia De Lebrusant Fernandez, Alessandro De Luca, Belinda De Simone, Francesca De Stefano, Luca Degrate, Zaza Demetrashvili, Andreas Kyriacou Demetriades, Dzemail Smail Detanac, Agnese Dezi, Giuseppe Di Buono, Isidoro-D. I. Carlo, Pierpaolo Di Lascio, Marcello Di Martino, Salomone Di Saverio, Bogdan Diaconescu, Jose J. Diaz, Rigers Dibra, Evgeni Nikolaev Dimitrov, Vincenza Paola Dinuzzi, Sandra Dios-Barbeito, Jehangir Farman Ali Diyani, Agron Dogjani, Maurizio Domanin, Mario D’Oria, Virginia Duran Munoz-Cruzado, Barbora East, Mikael Ekelund, Gerald Takem Ekwen, Adel Hamed Elbaih, Muhammed Elhadi, Natalie Enninghorst, Mairam Ernisova, Juan Pablo Escalera-Antezana, Sofia Esposito, Giuseppe Esposito, Mercedes Estaire-Gãmez, Camilla Nikita Farã, Roser Farre, Francesco Favi, Luca Ferrario, Antonjacopo Ferrario di Tor Vajana, Claudia Filisetti, Francesco Fleres, Vinicius Cordeiro Fonseca, Alexander Forero-Torres, Francesco Forfori, Laura Fortuna, Evangelos Fradelos, Gustavo P. Fraga, Pietro Fransvea, Simone Frassini, Giuseppe Frazzetta, Isabella Frigerio, Maximos Frountzas, Mahir Gachabayov, Rita Galeiras, Belen Matãas Garcãa, Alain Garcia Vazquez, Simone Gargarella, Ibrahim Umar Garzali, Wagih Mommtaz Ghannam, Faiz Najmuddin Ghazi, Lawrence Marshall Gillman, Rossella Gioco, Alessio Giordano, Luca Giordano, Carlo Giove, Giorgio Giraudo, Mario Giuffrida, Michela Giulii Capponi, Emanuel Gois, Carlos Augusto Gomes, Felipe Couto Gomes, Ricardo Alessandro Teixeira Gonsaga, Emre Gonullu, Jacques Goosen, Tatjana Goranovic, Raquel Gracia-Roman, Giorgio Maria Paolo Graziano, Ewen Alexander Griffiths, Tommaso Guagni, Dimitar Bozhidarov Hadzhiev, Muad Gamil Haidar, Hytham K. S. Hamid, Timothy Craig Hardcastle, Firdaus Hayati, Andrew James Healey, Andreas Hecker, Matthias Hecker, Edgar Fernando Hernandez Garcia, Adrien Montcho hodonou, Eduardo cancio huaman, Martin Huerta, Aini Fahriza Ibrahim, Basil Mohamed Salabeldin Ibrahim, Giuseppe Ietto, Marco Inama, Orestis Ioannidis, Arda Isik, Nizar Ismail, Azzain Mahadi Hamid Ismail, RUHI Fadzlyana Jailani, Ji Young Jang, Christos Kalfountzos, Sujala Niatarika Rajsain Kalipershad, Emmanouil Kaouras, Lewis Jay Kaplan, Yasin Kara, Evika Karamagioli, Aleksandar Karamarkovia, Ioannis Katsaros, Alfie J. Kavalakat, Aristotelis Kechagias, Jakub Kenig, Boris Juli Kessel, Jim S. Khan, Vladimir Khokha, Jae Il Kim, Andrew Wallace Kirkpatrick, Roberto Klappenbach, Yoshiro Kobe, Efstratios Kofopoulos Lymperis, Kenneth Yuh Yen Kok, Victor Kong, Dimitris P. Korkolis, Georgios Koukoulis, Bojan Kovacevic, Vitor Favali Kruger, Igor A. Kryvoruchko, Hayato Kurihara, Akira Kuriyama, Aitor Landaluce-Olavarria, Pierfrancesco Lapolla, Leo Licari, Giorgio Lisi, Andrey Litvin, Aintzane Lizarazu, Heura Llaquet Bayo, Varut Lohsiriwat, Claudia Cristina Lopes Moreira, Eftychios Lostoridis, Agustãn Tovar Luna, Davide Luppi, Gustavo Miguel Machain V., Marc Maegele, Daniele Maggiore, Stefano Magnone, Ronald V. Maier, Ronald V. Maier, Piotr Major, Mallikarjuna Manangi, andrea manetti, Baris Mantoglu, Chiara Marafante, Federico Mariani, Athanasios Marinis, EvandroAntonio Sbalcheiro Mariot, Giuseppe Roberto Marseglia, Aleix Martãnez-Pãrez, Gennaro Martines, Aleix Martinez Perez, Costanza Martino, Pietro Mascagni, Damien Massalou, Belãn Matãas-Garcãa, Gennaro Mazzarella, Giorgio Mazzarolo, Renato Bessa Melo, Fernando Mendoza-Moreno, Serhat Meric, Jeremy Meyer, Luca Miceli, Nikolaos V. Michalopoulos, Flavio Milana, Andrea Mingoli, Tushar S. Mishra, Muyed Mohamed, Musab Isam Eldin Abbas Mohamed, Ali Yasen Mohamedahmed, Mohammed Jibreel Suliman Mohammed, Rajashekar Mohan, Ernest E. Moore, Dieter Morales-Garcia, Mãns Muhrbeck, Francesk Mulita, Sami Mohamed Siddig Mustafa, Edoardo Maria Muttillo, Mukhammad David Naimzada, Pradeep H. Navsaria, Ionut Negoi, Luca Nespoli, Christine Nguyen, Melkamu Kibret Nidaw, Giuseppe Nigri, Ioannis Nikolopoulos, Donal Brendan O’Connor, Habeeb Damilola Ogundipe, Cristina Oliveri, Stefano Olmi, Ernest Cun Wang Ong, Luca Orecchia, Aleksei V. Osipov, Muhammad Faeid Othman, Marco Pace, Mario Pacilli, Leonardo Pagani, Giuseppe Palomba, Desire’ Pantalone, Arpad Panyko, Ciro Paolillo, Mario Virgilio Papa, Dimitrios Papaconstantinou, Maria Papadoliopoulou, Aristeidis Papadopoulos, Davide Papis, Nikolaos Pararas, Jose Gustavo Parreira, Neil Geordie Parry, Francesco Pata, Tapan Patel, Simon Paterson-Brown, Giovanna Pavone, Francesca Pecchini, Gianluca Pellino, Maria Pelloni, Andrea Peloso, Eduardo Perea del Pozo, Rita Goncalves Pereira, Bruno Monteiro Pereira, Aintzane Lizarazu perez, Teresa Perra, Gennaro Perrone, Antonio Pesce, Lorenzo Petagna, Giovanni Petracca, Vorapong Phupong, Biagio Picardi, Arcangelo Picciariello, Micaela Piccoli, Daniele Piccolo, Edoardo Picetti, Emmanouil Pikoulis Pikoulis, Tadeja Pintar, Giovanni Pirozzolo, Francesco Piscioneri, Mauro Podda, Alberto Porcu, Francesca Privitera, Clelia Punzo, Silvia Quaresima, Martha Alexa Quiodettis, Niels Qvist, Razrim Rahim, Filipe Ramalho de Almeida, Rosnelifaizur Bin Ramely, Huseyin Kemal Rasa, Martin Reichert, Alexander Reinisch-Liese, Angela Renne, Camilla Riccetti, Maria Rita Rodriguez-Luna, Daniel Roizblatt, Andrea Romanzi, Luigi Romeo, Francesco Pietro Maria Roscio, Ramely Bin Rosnelifaizur, Stefano Rossi, Andres M. Rubiano, Elena Ruiz-Ãšcar, Boris Evgeniev Sakakushev, Juan Carlos Salamea, Ibrahima Sall, Lasitha Bhagya Samarakoon, Fabrizio Sammartano, Alejandro Sanchez Arteaga, Sergi Sanchez-Cordero, Domenico Pietro Maria Santoanastaso, Diego Sasia, Norio Sato, Artem Savchuk, Robert Grant Sawyer, Giacomo Scaioli, Dimitrios Schizas, Simone Sebastiani, Barbara Seeliger, Helmut Alfredo Segovia Lohse, Charalampos Seretis, Giacomo Sermonesi, Mario Serradilla-Martin, Vishal G. Shelat, Sergei Shlyapnikov, Theodoros Sidiropoulos, Romeo Lages Simoes, Leandro Siragusa, Boonying Siribumrungwong, Mihail Slavchev, Leonardo Solaini, gabriele soldini, Andrey Sopuev, Kjetil Soreide, Apostolos Sovatzidis, Philip Frank Stahel, Matt Strickland, Mohamed Arif Hameed Sultan, Ruslan Sydorchuk, Larysa Sydorchuk, Syed Muhammad Ali Muhammad Syed, Ali Muhammad Syed, Luis Tallon-Aguilar, Andrea Marco Tamburini, Nicolò Tamini, Edward C. T. H. Tan, Jih Huei Tan, Antonio Tarasconi, Nicola Tartaglia, Giuseppe Tartaglia, Dario Tartaglia, John Vincent Taylor, Giovanni Domenico Tebala, Ricardo Alessandro Teixeira Gonsaga, Michel Teuben, Alexis Theodorou, Matti Tolonen, Giovanni Tomasicchio, Adriana Toro, Beatrice Torre, Tania Triantafyllou, Giuseppe Trigiante Trigiante, Marzia Tripepi, Julio Trostchansky, Konstantinos Tsekouras, Victor Turrado-Rodriguez, Roberta Tutino, Matteo Uccelli, Petar Angelov Uchikov, Bakarne Ugarte-Sierra, Mika Tapani Ukkonen, Michail Vailas, Panteleimon G. Vassiliu, Alain Garcia Vazquez, Rita Galeiras Vazquez, Juan Ezequiel Verde, Juan Manuel Verde, Massimiliano Veroux, Jacopo Viganò, Ramon Vilallonga, Diego Visconti, Alessandro Vittori, Maciej Waledziak, Tongporn Wannatoop, Lukas Werner Widmer, Michael Samuel James Wilson, Ting Hway Wong, Sofia Xenaki, Byungchul Yu, Steven Yule, Sanoop Koshy Zachariah, Georgios Zacharis, Claudia Zaghi, Andee Dzulkarnaen Zakaria, Diego A. Zambrano, Nikolaos Zampitis, Biagio Zampogna, Simone Zanghã, Konstantinos Zapsalis, Fabio Zattoni, Monica Zese, Silvia Pãrez Farre, Boyko Tchavdarov Atanasov, Veronica Pegoraro, Maristella Zantedeschi, Elisa Reitano, Erica Pizzocaro

**Affiliations:** 1 Department of Clinical, Diagnostic and Pediatric Sciences, University of Pavia, Via Alessandro Brambilla, 74, 27100 Pavia, PV Italy; 2 IRCCS Policlinico San Matteo Foundation, General Surgery, Pavia, Italy; 3 Department of Management, Ca’ Foscari University of Venice, Venice, Italy; 4 Bufalini Hospital, AUSL Romagna, Cesena, Italy; 5 Division of Trauma and Acute Care Surgery, Scripps Memorial Hospital La Jolla, La Jolla, CA USA; 6 Department of HPB Surgery, Pederzoli Hospital, Peschiera del Garda, Italy; 7 Department of Economics, Ca’ Foscari University of Venice, Venice, Italy; 8 Department of Economics and Management, University of Pavia, Pavia, Italy; 9 Department of Surgery, Careggi University Hospital, Florence, Italy; 10 General Direction, ASL BAT (Health Agency), Andria, Italy; 11 Department of Political and Social Sciences, University of Pavia, Pavia, Italy; 12 Bureau of the Presidency, IRCCS Policlinico San Matteo Foundation, Pavia, Italy; 13 Department of Surgery, Spaarne Gasthuis, Hoofddorp, The Netherlands; 14 Harvard Medical School, Boston, MA USA; 15 Division of Trauma, Emergency Surgery, and Surgical Critical Care, Massachusetts General Hospital, Boston, MA USA; 16 Department of Surgery, University of Florida Health, Gainesville, FL USA

**Keywords:** Shared decision-making, Clinical decision-making, Patient-centric care, Trauma and emergency surgery, Survey

## Abstract

**Background:**

Shared decision-making (SDM) between clinicians and patients is one of the pillars of the modern patient-centric philosophy of care. This study aims to explore SDM in the discipline of trauma and emergency surgery, investigating its interpretation as well as the barriers and facilitators for its implementation among surgeons.

**Methods:**

Grounding on the literature on the topics of the understanding, barriers, and facilitators of SDM in trauma and emergency surgery, a survey was created by a multidisciplinary committee and endorsed by the World Society of Emergency Surgery (WSES). The survey was sent to all 917 WSES members, advertised through the society’s website, and shared on the society’s Twitter profile.

**Results:**

A total of 650 trauma and emergency surgeons from 71 countries in five continents participated in the initiative. Less than half of the surgeons understood SDM, and 30% still saw the value in exclusively engaging multidisciplinary provider teams without involving the patient. Several barriers to effectively partnering with the patient in the decision-making process were identified, such as the lack of time and the need to concentrate on making medical teams work smoothly.

**Discussion:**

Our investigation underlines how only a minority of trauma and emergency surgeons understand SDM, and perhaps, the value of SDM is not fully accepted in trauma and emergency situations. The inclusion of SDM practices in clinical guidelines may represent the most feasible and advocated solutions.

## Background

Trauma and emergency surgery teams include a group of specialists (comprising surgeons, emergency physicians, anesthesiologists, and nurses, among others) cooperating to provide patients with high-quality care. Such professionals operate under challenging circumstances, high stress, and time pressures. They often have little awareness of the trauma’s causes, the patients’ identities, current conditions, and care preferences [1]. While team dynamics appear fundamental to ensuring the best quality of care from a patient-centric perspective, knowledge translation and sharing processes [2, 3] are essential to patient-centered care and the inviolable patient-physician relationship [4]. Indeed, the clinical team might lack time to examine the various possibilities and treatment options with the patients, including prognostic information [5, 6].

The value of shared decision-making (SDM) in patient-centered care is well recognized in the context of contemporary healthcare. Healthcare professionals and patients are encouraged to engage in SDM to jointly make decisions, considering the best available evidence and the patients’ values and treatment choices. SDM is anticipated to improve patient treatment compliance and, consequently, health outcomes. In particular, SDM is the most advantageous choice for judgments that must consider the patient’s preferences and wishes. When two or more comparable treatment options are available, healthcare professionals should support the patient in selecting his or her best option, depending on how each patient rates the benefits and hazards of each choice [7]. Therefore, SDM stands as a pillar of patients’ autonomy, and clinicians have the moral and ethical duty to support patients in making decisions that embrace their values and priorities [8]. Still, engaging in effective SDM practices is not a surgical care panacea. Barriers may emerge due to a gap in the clinical knowledge between the patient and the physician, the feelings and concerns that the patient may have, the complexity of interactions among diagnoses and treatments, and the lack of time or training to conduct a fruitful discussion with the patient and the family or caregivers. This means that both clinicians and patients should find effective ways and tools to translate and share knowledge, despite their differences in terms of backgrounds, medical mastery, concerns, and doubts [3, 9]. The recent clinical literature has highlighted the relevance of adequate facilitators to support such a practice [2, 10], including the role of non-technical skills in improving communication [5, 11, 12].

Recent experiences and studies in trauma and emergency settings have underlined how the implementation and measurement of SDM are complex [8]. Indeed, sometimes the patient’s life may be in danger, leaving little time to make a clinical decision. Still, other times, conditions may allow physicians hours or more before the treatment begins, giving patients time to learn about the potential alternatives and make an informed decision about the next medical steps. The surgical literature [13] has stressed the benefits of applying SDM in trauma and emergency surgery, including better clinical outcomes by enhancing the quality of patient’s recovery [14], better managing the patient’s expectations [15], and limiting surgical interventions when they are not necessary [16]. Benefits can also be gathered from the hospital’s organization, as SDM provides better patient management and flow [17], stimulating patient-centric care [18] and leading to patient empowerment and co-production practices [19, 20]. All in all, when SDM is employed in trauma and emergency settings, patients and their families enjoy a better hospital experience [21] while physicians comply with ethics and moral norms [22]. Training and counseling appear as the most common facilitators for the effective implementation of SDM in trauma and emergency settings [21, 23]. Time and resource limitations make it difficult for the in-charge physician to have a fruitful conversation with the patient. When the patient load is heavy, there is little opportunity to spend time with a single patient who needs to understand and decide from a range of clinical alternatives.

Starting from these premises and research gaps, the paper aims to deepen the barriers, facilitators, and dynamics of SDM in trauma and emergency settings, by employing a multi-national survey endorsed by the World Society of Emergency Surgery (WSES).

## Methods

### Design and setting

Our exploratory study of the international trauma and emergency surgeons’ community used a population-based online questionnaire to gather demographic, knowledge, and practice-based information regarding their SDM understanding and dynamics. The online questionnaire was conducted in English through Google Forms [4, 6], and followed the Checklist for Reporting Results of Internet E-Surveys (CHERRIES), as reported in Appendix 1 [24].

A steering committee within the WSES was created, involving a multidisciplinary panel of practitioners and scholars in the fields of trauma and emergency surgery, healthcare management, innovation, and organization science. No Institutional Review Board (IRB) approval was needed, as non-interventional studies do not necessarily require approval by an ethics committee. The survey participants were exclusively clinicians who decided to participate voluntarily. No significant identifying information about the participants is possible. The study was conducted following the principles of the Declaration of Helsinki.

Starting from a review of the literature, a research protocol was conceived and shared by the principal investigators (LC and FDM) with the steering committee. The leading references to create the protocol and the survey structure were gathered from Woltz et al. [8], Mathijssen et al. [7], Cobianchi et al. [4], and Dal Mas et al. [2]. Before the initiative’s official launch, the research protocol and the online survey were reviewed by the steering committee and filled in by a sample of surgeons to avoid mistakes.

The survey was launched at the end of November 2021 and remained open until mid-August 2022. An e-mail invitation to participate in the initiative was sent out within the WSES newsletter to all 917 WSES members and disseminated through the society’s website and Twitter profile. Moreover, an e-mail invitation was sent out to the mailing list of the Team Dynamics Study Group [4, 6]. Four reminders followed through the same channels. Although WSES membership was not a prerequisite for enrollment, we expect that most of the participants come from the 917 WSES members to whom the research initiative was advertised, obtaining, therefore, a response rate close to 70%.

The invitation e-mail comprised detailed information about the initiative’s rationale and aims, the expected duration (approximately 10 min), and the opportunity of signing up in the Team Dynamics Study Group to continue investigating and sharing the findings. The participants’ identities were kept anonymous. The research protocol and the investigators’ names were kept confidential as well.

### Survey

The first group of questions aimed at understanding the participants’ characteristics. The same questions were gathered from the previous Team Dynamics investigation [4, 6], and they included gender, the number of years of experience in trauma/emergency surgery, the kind of institution (academic vs non-academic), the country, the position held, the eventual participation within a trauma team (institutionalized or not, and of which kind), the type of trauma leader, the educational courses attended, and the presence of diverse team members.

The second group of questions aspired to understand trauma and emergency surgeons’ perception and knowledge of SDM by employing a yes/no question and an open question, following Woltz et al. [8].

The third group of questions wanted to investigate the frequency and perception of SDM starting from a list of items gathered from Woltz et al. [8] and Mathijssen et al. [7] to be rated on a 5-point Likert scale.

The fourth and last group of questions aimed at exploring the barriers and facilitators to SDM, to be rated on a 5-point Likert scale. More specifically, barriers were gathered from the study of Mathijssen et al. [7], while facilitators were inspired from the original list of 32 items mentioned by Dal Mas et al. [2], which were later grouped into nine categories, as also reported by other investigations [4].

The survey’s questions related to SDM are reported in Appendix 2.

### Statistical analysis

Descriptive statistical analysis was conducted using the software R [25].

Manual coding was also employed concerning the qualitative questions. Concerning the understanding of SDM, participants were then asked to provide a definition of SDM through an open question. Results were manually coded by two researchers (LC and FDM), who rated each statement as concordant, discordant, or inconclusive, following the analysis of Woltz et al. [8] and Cobianchi et al. [4]. The same methodology was applied concerning the situations or conditions where SDM could be used. Two researchers (LC and FDM) coded all the statements to group them into meaningful categories.

## Results

### Participants

The questionnaire was filled in by 650 surgeons. Participants came from 71 countries on the five continents. Still, the sample was not equally distributed, with most surgeons coming from Europe (477, 73%) and especially Italy (251, 39%). The ten countries with the highest number of participants globally accounted for 465 respondents (72%).

The sample was made up of 118 female surgeons (18%), 531 males (82%), and one participant preferring not to disclose their gender. Surgeons had a range of 1–35 years of experience in the field, with a mean of 12. Most participants came from academic institutions (499, 77% of the sample), with 540 of them officially part of an emergency surgery team (83%). The roles declared varied, with the majority of surgeons being senior consultants (233, 36%). One hundred and fourteen (18%) were departmental heads.

Table 1 reports the descriptive statistics about the participants and institutions involved in the study, while Table 2 highlights some statistics about the number of respondents according to their locations.

**Table 1:** Descriptive statistics about surgeons and institutions participating in the investigation

Item	Number	%
Participants	650	100.00
Males	531	81.69
Females	118	18.15
Prefer not to answer	1	0.15

	Mean	Standard deviation
Years of experience	12.32	8.42
Min	1	
Max	36	

	Number	%
Kind of Institution	650	100.00
Academic	499	76.77
Non-academic	151	23.23
Role/position	650	100.00
Senior Consultant	233	35.85
Board-certified surgeon	179	27.54
Resident	124	19.08
Division chief or head	114	17.54
Part of an emergency surgery team	650	100.00
Yes	540	83.08
No	110	16.92

**Table 2:** Number of respondents according to their location

Total participants	650	100%
*Number of countries*	71	
*Continents*	5	100
Europe	477	73
Asia	85	13
America	62	10
Africa	22	3
Oceania	4	1
*Ten most present countries*	465	72
Italy	251	39
Greece	45	7
Spain	37	6
United Kingdom	37	6
United States	26	4
Turkey	16	2
Malaysia	15	2
France	14	2
Brazil	13	2
Ukraine	11	2

### Understanding of SDM

Regarding the understanding of the concept of SDM, surgeons were first asked if they were familiar with the term. Four hundred and eighty-four of them (74%) replied they were, and the remaining 166 (26%) declared they were not. As specified, each given statement was rated as concordant, discordant, or inconclusive.

To be rated as concordant, definitions needed to stress the concept of surgeons (or multidisciplinary trauma or emergency team) involving the patient in the clinical decisions. Interestingly, less than half of the participants (290, 45% of the sample) provided definitions that could be rated as concordant according to the abovementioned criterion. 93 participants (14% of the sample) gave responses that were incomplete, showing only a partial view of the phenomenon, being so rated as inconclusive. The remaining 267 surgeons (41%) gave answers that were not fitting the general definition of SDM. Interestingly, while some participants declared that they did not know what SDM meant, or did not provide any concrete answers (73, equal to 11%), most (194, 30% of the sample) stressed the multidisciplinary aspect of emergency care and the need to decide within the clinical team, not even mentioning the possibility to include the patient in the picture.

Table 3 reports some examples of answers that were rated as concordant, inconclusive, and discordant [4, 8].

**Table 3:** Examples and ways of rating the given answers to the question: What is your understanding of SDM?

Rated as	Given answer	Reason for rating
Concordant	“SDM is a joint process in which a healthcare professional works together with a person to reach a decision about care.”	The definition recalls the idea of involving the patient in the clinical decisions
	“Choose the best therapeutic approach for the patient by sharing it with the patient and family members.”	
	“Part of a more comprehensive patient-centered/focused health care. As summary, physician/surgeon and patient share decision-making process, working collaboratively to define the best patient journey putting her/him in the center and always based in the most relevant evidence.”	
	“A process where healthcare professionals work together with patients with/without families to reach a decision about the patient’s care.”	
	“Surgeon and patient collaboratively choose the most appropriate treatment for the patient, after adequate information about possible treatments (surgeon) and individual necessities/beliefs (patient) are shared reciprocally.”	
	“It is a process in which the patient and doctors participate in the medical decision-making process and agree on treatment decisions.”	
	“Taking a decision based on shared opinion by the patient and a team of physicians. Both sides should be carefully informed and reach to a decision that benefits the patient.”	
	“Discussion adn condivision, between either patient and physician, about the therapeutics possibilities.”	
Inconclusive	“Collaborative decision-making.”	It is unclear with whom such decision-making should occur
	“Collaborative process for shared decisions.”	
	“Sharing of diagnostic and therapeutic choices.”	
	“Everyone has a full role in the decision-making process.”	
	“Inform and take consent from patients.”	Although informed consent is relevant and may represent a tool for SDM, it is not enough to qualify as SDM
	“One or some best decision for a particular patient.”	It is unclear if and to what extent the patient participates in the process or agrees to the medical decision
	“Sufficient theoretically but quite poor practically.”	Not clear
Discordant	“Share decision with other members of the team to choose the best treatment.”	The patient’s dimension is missing
	“It is a collaborative process as healthcare professionals work together to make a decision about care.”	
	“Sharing decisions made by different specialities that members of an ER team.”	
	“Multidisciplinary collaboration in order to get the good choices for the patient.”	
	“When we should go in the OR and make maybe more radical procedures.”	
	“A decision taken after a multidisciplinary approach.”	
	“A surgeon leads, facilitates other specialist referrals, inputs referring specialist decisions, leads surgeon together with other specialists plans and prioritise management plan.”	
	“Find the best therapeutic indication shared by the team.”	
	“Senior team consultation prior to decision-making”	
	“Following guidelines and protocols, each member has a specific role and knows what to do.”	Guidelines and protocols can recommend SDM processes; however, they do not represent the key point
	“Leadership”	Although leadership may facilitate SDM, it still misses the key point of involving the patient

### Engaging in SDM

Surgeons were asked to rate 15 items gathered from the studies of Woltz et al. [8] and Mathijssen et al. [7] using a 5-point Likert scale where 1 meant “not important” and 5 “very important.”

Although all 15 items got a mean evaluation of over 3.78, some of them got a major agreement. Findings reveal a significant relationship between an SDM mindset and a patient-centric view. In particular, surgeons recognized the value of informing the patient about the pros and cons of the chosen treatment plan (mean 4.62, with a standard deviation of 0.67), and explaining the chance of those favorable or adverse outcomes happening. Decision-making seems a very relevant concept to trauma and emergency surgeons, as they recognized the importance of sharing the fact that a decision has to be made.

Among the less rated items, we underline the need to spend time investigating the patient’s preferences (mean 3.78, with a standard deviation of 1). Interestingly, there was one more similar item in the list, reported as “Understanding the patient’s references,” which got a higher evaluation (mean 4.21, with a standard deviation of 0.89). The less rated item was about asking the patient to bring someone (maybe a family member or caregiver) to the consultation (mean 3.78, with a standard deviation of 1.03).

Table 4 reports the results related to the relevant items to SDM.

**Table 4:** Relevant items to SDM

Item	Mean	SD
Informing the patients about all the pros and cons of the chosen treatment plan	4.62	0.67
Informing the patient about the chance of these pros and cons	4.53	0.69
Informing the patient that a decision has to be made	4.53	0.71
Explaining why some treatment options should be preferred	4.47	0.75
Explaining all the available treatment options	4.45	0.78
Explaining to the patient that his/her opinion is important in making the decision	4.27	0.88
Understanding the patient’s preferences	4.21	0.89
Letting the patient decide after informing him/her	4.17	0.93
Allowing the patient to co-decide the treatment	4.16	0.91
Making clinical decisions which are aligned to the patient’s preferences	4.00	0.97
Letting the patient repeat the given information	3.98	1.02
Giving information in more ways than only verbally (e.g., leaflet, website)	3.93	1.05
Allowing the patient time by making the decision in a second consultation	3.87	0.97
Spending time to investigate about the patient’s preferences	3.78	1.00
Asking the patient to bring someone to the consultation	3.78	1.03

The survey also included a list of potential barriers that could make it challenging for surgeons to engage in SDM practices. Those items were gathered from the investigation of Mathijssen et al. [7], to be rated using a 5-point Likert scale where 1 meant “not important” and 5 “very important.”

The participants stressed that in emergency contexts, decisions often need to be taken in within a very short period of time (mean 4.11, standard deviation 0.88). They also claimed that emergency and trauma teams collaborate successfully (mean 3.83, standard deviation 0.94). Surgeons denied that SDM practices might be in contrast with clinical guidelines (mean 2.16, standard deviation 1.2). Therefore, that would not represent a barrier to its practical application.

Full results and ratings are reported in Table 5.

**Table 5:** Barriers to SDM

Item	Mean	SD
Sometimes decisions are urgent and have to be made right away	4.11	0.88
Multidisciplinary emergency teams collaborate successfully with each other	3.83	0.94
Sometimes there are communication issues (e.g., language barriers)	3.66	1.03
Patients prefer to say: “You decide” or “Do what you think is best, doc”	3.60	1.02
Patients lack knowledge of treatment options	3.57	1.08
There are many other things demanding the attention of healthcare professionals	3.44	1.04
Healthcare professionals forget to apply SDM as it is not part of the routine	3.34	1.08
Healthcare professionals feel that they lack knowledge about what SDM entails	3.21	1.06
Several colleagues do not believe in SDM	3.21	1.17
The inter-professional collaboration is inadequate (e.g., poor communication within the team)	3.10	1.09
Time should be dedicated to other tasks than SDM	2.99	1.12
SDM causes patients to question the expertise of healthcare professionals	2.83	1.25
There is not enough time to apply SDM (e.g., consultation times are too short)	2.82	1.20
Some treatment options are too expensive to be taken into account	2.74	1.23
SDM is incompatible with clinical practice guidelines	2.16	1.20

### Situations or diagnoses in trauma and emergency surgery suitable for SDM

Through an open question, participants were asked to name any situation and/or clinical condition in which SDM practices may be successfully applied. As anticipated, statements were coded and grouped into meaningful categories.

Part of the sample named some situations. For instance, 84 participants claimed that any situation may be suitable for SDM practices if the patient or a substitute (namely, a family member or caregiver) is available to discuss with the physician. 82 surgeons named some trauma situations, with the patient still able to interact with the clinical team. 20 participants were not able to provide examples. Interesting enough, 11 of them claimed that no situation in trauma or emergency contexts would be suitable for SDM practices.

The other part of the sample preferred to name some specific conditions or diagnoses. Among the most rated, we can recall appendicitis/diverticular disease (116), acute abdomen (54), non-urgent oncological issues (37), acute cholecystitis (36), and bowel obstruction (31).

The full list of items is reported in Table 6.

**Table 6:** Situations/diagnoses suitable for SDM

*Situations*	
Any if the patient or substitute is available	84
Some trauma situations [in general terms]	82
No reply provided	20
Elderly patients with multiple comorbidities	13
No situations are suitable for SDM	11
All situations in their diagnostic/exploration phase	7
*Diagnoses*	
Appendicitis/diverticular disease	116
Acute abdomen	54
Oncology in general [no colon]	37
Acute cholecystitis	36
Bowel obstruction	31
Colon cancer	30
Fracture	22
Splenectomy	19
Amputation	10
Gastrointestinal bleeding	10
Perforation [various organs]	10
Blunt thoracic aortic injury	10
Acute biliary pancreatitis	8
Neurosurgical conditions	8
Palliative care	7
Stab wound	4
Strangulated hernia	4
Other examples [less than two mentions]	17
Total	650

### Facilitators

Surgeons were asked about the facilitators that could support SDM practices. Those which recorded the highest importance were training (mean 4.3/5), time to engage patients (mean 4.19/5), clinical guidelines and cases (mean 4.18/5), and cultural competence (mean 4.17/5). The less rated item was that of electronic medical records (mean 3.74/5). Results are reported in Table 7.

**Table 7:** Facilitators to SDM

Item	Mean	SD
Training	4.30	0.83
Time spent to engage patients	4.19	0.90
Clinical guidelines and cases	4.18	0.86
Cultural competence	4.17	0.89
Multidisciplinary committees and meetings	4.15	0.90
Non-technical skills	4.04	1.01
Networking and international experiences	3.97	0.95
Publications	3.83	0.96
Mobile electronic medical records and online tools, including telemedicine	3.74	1.11

## Discussion

SDM represents one of the pillars of the modern healthcare scenario [26]. Scholars, policymakers, and healthcare institutions advocate the right of the patient to actively participate in clinical decisions, along with the physicians or medical team in charge. The advantages of SDM are numerous [13], and they recall better satisfaction and hospital experience for the patient [14], and better alignment with the chosen treatment [19, 20].

While in some medical specialities, like oncology, SDM is widely applied [27, 28], previous studies have underlined difficulties in engaging in SDM practices in emergency and trauma contexts [8]. Suppose some of such barriers refer to the fact that sometimes patients’ lives are in danger, or the patient is unconscious and maybe his/her identity is unknown. Still, there may be some emergency and trauma situations in which SDM can be applied, as there may be hours or more time available before the treatment begins.

Our investigation, endorsed by the WSES, had to aim to deepen the dynamics, understanding, barriers, and facilitators of SDM practices, enquiring trauma, and emergency surgeons. Results are in line with the previous literature [8], and some interesting findings emerge.

While most of the surgeons declared that they were familiar with the term “SDM,” open responses about its meaning depict a completely different image. Indeed, only 45% of the participants were able to provide a definition that matched the concept of the patient being involved in the clinical decisions. More than 11% of our participants had no idea about what SDM entailed, and the 30% of them had a completely different (and wrong) meaning in mind. Hundreds of surgeons saw SDM as the clinical trauma or emergency team members co-deciding and discussing the treatment options for the patient. The word “multidisciplinary” was named several times to stress the effort to join forces by enquiring colleagues with different backgrounds or expertise. Still, the process was seen as “doctors-only.” Interestingly, several surgeons name the need to find a solution “in the best patient’s interests” to underline how much physicians care about the best possible outcome for their patients. Still, it seems like it is neither doable nor useful to engage the patient and the family in the decision-making process. Clinical teams know how to do their job, and together they can reach the best clinical decision according to the situation. All the efforts are, indeed, dedicated to adequate knowledge translation and communication processes within the team, involving also non-technical skills like leadership [1, 4].

Similar results can also be gathered when deepening the topics of engaging in SDM processes in terms of practices and barriers. Surgeons generally recognize the importance of informing the patient about a specific treatment option’s advantages and hazards and aligning (whenever possible) the treatment to the patient’s values and wishes. However, surgeons seem less available to “investigate” such preferences when they are not transparent or maybe when a communication effort is required to engage with the patient. Barriers are defined mainly in the lack of time that often characterizes trauma and emergency contexts. Still, when enquired about possible situations or conditions when SDM might be successfully applied, surgeons did provide several examples. Among the facilitators to support such practices, participants named training but also clinical guidelines, while surgeons seem to have less trust in technological devices and online tools.

Our investigation underlined how trauma and emergency surgeons seem more concentrated on making things work within their teams rather than engaging in dialogues with their patients, with a lack of an in-depth understanding of the benefits of such a practice. Such results are not surprising, not only as emergency and trauma situations often need to be managed within minutes, but also when we consider the debate going on in such a specific surgical speciality. Indeed, team dynamics are deemed crucial to reaching the best clinical outcomes, and much effort (also from scientific societies like the WSES) has been concentrated on topics like communication, non-technical skills, etc. It seems like surgeons see the best value in making their teams work smoothly than looking at what is happening outside.

As said, the literature has underlined how engaging in SDM practices requires adequate tools and facilitators, as the competencies and emotional gap between the patient and the physician may be broad [29]. Still, a patient-centric philosophy of care cannot leave such topics behind, even when it comes to challenging situations like those connected to trauma or emergencies. While surgeons strongly believe in training, they also rely on clinical guidelines, which should encompass such principles and values even when reporting tough clinical situations or conditions. In this perspective, the role of scientific societies like the WSES is again crucial to take the lead in stimulating a paradigm shift, in which team dynamics are essential, but so are the relationships with the patients. In such a view, other team members may support surgeons in successfully dealing with such dynamics, for example, nurses, who usually spend more time with patients and their families [30–33].

Last but not least, an open issue arises about using technologies to support SDM practices. Do such technological tools represent minor or weak support for SDM, or is there a problem connected to the digital culture among healthcare professionals? The fact that such an item records the highest standard deviation (1.11) highlights a significant divergence in opinions. Therefore, such an issue deserves further investigation in future studies.

### Limitations

Although our sample is numerous, with 650 participants, it is not equally distributed. Indeed, most participants work in Europe and, more specifically, in Italy. The specific situation of the Italian and European contexts (including the features of National Healthcare Systems) may have biased some of our results. Moreover, most participants are men and belong to academic institutions. Again, such situations may have impacted some views or responses. Our limitations, along with the perceived interest of the international community on the topic of SDM, may stimulate new in-depth studies and investigations on such a relevant and up-to-date theme.

## Conclusion

In concluding our work, we should begin from the premise that inspired it. SDM represents a crucial and “hot” topic in today’s healthcare atmosphere, involving all medical specialities. Emergency and trauma contexts often represent challenging situations in which SDM may look difficult to apply. Surprisingly, only less than half of the inquired surgeons are familiar with the term and meaning of SDM. The 30% of the participants of our study wrongly identify SDM as multidisciplinary decision-making among medical team’s members, not seeing the value of involving the patient in the process. Modern patient-centered ethics sees a call for all medical professionals to find ways to engage patients in clinical decisions whenever possible. Such a call involves emergency and trauma surgeons as well.

Our results suggest the need for scientific societies like the WSES, undergraduate and postgraduate educational institutions, and healthcare managers and policymakers to stimulate an SDM culture, also through training courses and formalized guidelines. Therefore, our findings may be relevant to support practical actions.

## Data Availability

The datasets used and/or analyzed during the current study are available from the corresponding author upon reasonable request.

## Appendix 1

**Table:**

Item Category	Checklist Item	Explanation
Design	Survey design	See the survey protocol Designed in August 2021
IRB approval and informed consent process	IRB approval	Not required
	Informed consent	Not required
	Data protection	Not required
Development and pre-testing	Development and testing	Pre-testing done in September and October 2021
Recruitment process and description of the sample having access to the questionnaire	Closed survey	August 2022
	Contact mode	Newsletter sent on November 29th, 2021 https://www.wses.org.uk/news/decision-making-survey-a-wses-team-dynamics-study-group-initiative
	Advertising the survey	Social media post (Linkedin) on November 29^th^, 2021
Survey administration	Web/Email	Teamdynamics2021@gmail.com
	Context	WSES members
	Voluntary	Yes
	Incentives	Participation in the WSES Team Dynamics study group (see questions #0 & #0)
	Time/Date	Opened from November 29^th^ 2021, till August 18^th^ 2022
	Randomization of items	See the survey protocol
	Adaptive questioning	See the survey protocol
	Number of items	See the survey protocol
	Number of screens	Three screens
	Completeness check	YES
	Review step	NO
Response rates	Unique site visitor	Not available
	View rate	Not available
	Participation rate	70%
	Completion rate	100%
Preventing multiple entries from the same individual	Cookies used	Not available
	IP check	Not available
	Log file analysis	Not available
	Registration	Not available to ensure anonymity
Analysis	Handling of incomplete questionnaires	Not applicable
	Questionnaires submitted with an atypical timestamp	None
	Statistical correction	None

*Web survey link: https://forms.gle/WGzfNQ7GVPgFkQcKA

## Appendix 2

1. **Are you familiar with the term SDM?**
  ***Yes/No***
  1. Yes
  2. No

2. **What is your understanding of SDM?**
  ***open question***
3. **On a scale from 1 to 5, where 1 = not important and 5 = very important, how would you rate the following items?**
  *** Likert scale 1 to 5***
  1. Understanding the patient’s preferences
  2. Making clinical decisions which are aligned to the patient’s preferences
  3. Informing the patients about all the pros and cons of the chosen treatment plan
  4. Informing the patient about the chance of these pros and cons
  5. Informing the patient that a decision has to be made
  6. Allowing the patient time by making the decision in a second consultation
  7. Spending time to investigate about the patient’s preferences
  8. Letting the patient decide after informing him/her
  9. Asking the patient to bring someone to the consultation
  10. Explaining all the available treatment options
  11. Explaining why some treatment options should be preferred
  12. Allowing the patient to co-decide the treatment
  13. Explaining to the patient that his/her opinion is important in making the decision
  14. Letting the patient repeat the given information
  15. Giving information in more ways than only verbally (e.g., leaflet, website)

4. **On a scale from 1 to 5, where 1 = never and 5 = always, how frequently do you engage in SDM if a decision is suitable for this?**
  ***Likert scale 1 to 5***
5. **On a scale from 1 to 5, where 1 = strongly disagree and 5 = strongly agree, how would you rate the following statements?**
  ***Likert scale 1 to 5***
  1. Time should be dedicated to other tasks than SDM
  2. Sometimes decisions are urgent and have to me made right away
  3. SDM causes patients to question the expertise of healthcare professionals
  4. Healthcare professionals feel that they lack knowledge about what SDM entails
  5. Healthcare professionals forget to apply SDM as it is not part of the routine
  6. There are many other things demanding the attention of healthcare professionals
  7. Patients prefer to say: “You decide” or “Do what you think is best, doc”
  8. Patients lack knowledge of treatment options
  9. Sometimes there are communication issues (e.g., language barriers)
  10. The inter-professional collaboration is inadequate (e.g., poor communication within the team)
  11. Several colleagues do not believe in SDM
  12. Multidisciplinary emergency team collaborate successfully with each other
  13. SDM is incompatible with clinical practice guidelines
  14. There is not enough time to apply SDM (e.g., consultation times are too short)
  15. Some treatment options are too expensive to be taken into account.

6. **Please describe a situation or diagnosis in emergency surgery that you find suitable for SDM**
  ***open question***
7. **On a scale from 1 to 5, where 1 = not suitable and 5 = very suitable, which are the tools that, in your opinion, may facilitate shared decision-making?**
  ***Likert scale 1 to 5***
  1. Mobile electronic medical records and online tools, including telemedicine
  2. Training
  3. Networking and international experiences
  4. Multidisciplinary committees and meetings
  5. Publications
  6. Clinical guidelines and cases
  7. Time spent to engage patients
  8. Non-technical skills
  9. Cultural competence

## Abbreviations

SDM: Shared decision-making
WSES: World Society of Emergency Surgery
CHERRIES: Checklist for Reporting Results of Internet E-Surveys
IRB: Institutional Review Board
ER: Emergency Room
OR: Operating Room
